# Spatial distribution and multilevel analysis of factors associated with long-acting reversible contraceptive use among sexually active women of reproductive age in Nigeria

**DOI:** 10.1186/s13690-023-01110-6

**Published:** 2023-06-02

**Authors:** Obasanjo Afolabi Bolarinwa, Kobi V. Ajayi, Sylvester Reuben Okeke, Samuel Hailegebreal, Clifford Odimegwu

**Affiliations:** 1 Department of Public Health & Well-being, Faculty of Health, Medicine & Society, Chester, CH1 1SL UK; 2 Department of Public Health Medicine, School of Nursing and Public Health, University of KwaZulu-Natal, Durban, South Africa; 3 Department of Health Behavior, School of Public Health, Texas A&M University, College Station, TX 77843 USA; 4 Centre for Social Research in Health, UNSW Sydney, Sydney, Australia; 5 Department of Health Informatics, School of Public Health, College of Medicine and Health Sciences, Wachemo University, Hossana, Ethiopia; 6 Demography and Population Studies Programme, Schools of Public Health and Social Sciences, University of the Witwatersrand, Johannesburg, South Africa

**Keywords:** Long-acting reversible contraceptive use, Spatial analysis, Multilevel analysis, DHS, Nigeria

## Abstract

**Background:**

Long-acting reversible contraceptives (LARCs), including hormonal implants and intrauterine devices, are highly effective pregnancy prevention methods. Aside its advantages over other hormonal methods, LARCs are cost-effective, easy to maintain, and have a low risk of non-compliance-related method failure. Besides, LARCs are also relatively safe for all sexually active women in the postpartum or post-abortion period. However, despite its effectiveness, most sexually active women use other short-term methods, such as condoms and contraceptive pills, which are associated with high discontinuation rates. Thus, this study examines the spatial distribution and multilevel factors associated with LARC use among sexually active reproductive-age women in Nigeria.

**Methods:**

This is a cross-sectional analysis of a population-based study from the 2018 Nigeria Demographic Health Survey (NDHS). The NDHS is a nationally representative survey that collects data on socio-demographic characteristics, sexual and reproductive health-related indicators such as contraceptive use and child & maternal health. A sample of 3,978 sexually active reproductive-age women (15–49 years) in Nigeria was used in the analysis. Frequency distribution and spatial analysis of LARC use were displayed with tables and maps, respectively, while multilevel analysis at a 95% confidence interval (CI) and a p-value of less than 0.05 was used to determine factors associated with LARC use among the sample.

**Results:**

The spatial distribution of LARC use among sexually active women of reproductive age in Nigeria ranges between 20 and 34.8%. Fifteen of the 36 states (excluding the Federal Capital Territory, FCT) recorded low utilization of LARCs. These states include Adamawa, Lagos, Ogun, Enugu, Anambra, Imo, Abia, Rivers, Kogi, Taraba, Yobe, Gombe, Jigawa, Borno, and Kebbi. Besides, the likelihood of LARC use was lower among participants with a prior history of pregnancy termination [aOR = 0.62; 95%(CI = 0.48–0.80)] compared to their counterparts without pregnancy termination history. Also, participants with no fertility intention had a higher likelihood of using LARCs [aOR = 1.65; 95%(CI = 1.30–2.08)] compared to those with fertility intention. At the community level, women with higher socioeconomic status were less likely to use LARCs [aOR = 0.66; 95%(CI = 0.45–0.97)] compared to women with lower socioeconomic status.

**Conclusions:**

This study showed a relatively low utilisation of LARC among sexually active reproductive-age women in Nigeria. Notably, this low utilisation is also common in states that could be described as cosmopolitan, indicating a need for closer investigation to understand context-specific factors associated with LARC use. Population-specific family planning education and counselling for this population are important to address common misconceptions about LARCs in particular and modern contraceptive use in general.

**Table:** 

Text box 1. Contributions to the literature	
• Research has shown how long-acting reversible contraceptives (LARCs) among women efficiently avert child and maternal adverse health outcomes. However, there is no specific study in Nigeria that has examined the use of LARCs among sexually active reproductive-age women using spatial analysis and multilevel analysis. • This study’s results showed an unequal distribution of LARC use across the regions in Nigeria. Cosmopolitan states were found to have low LARC utilisation. • Women with a prior history of pregnancy termination were less likely to use LARCs, indicating a need for closer investigation to understand context-specific factors associated with LARC use in these regions.	

## Background

Long-acting reversible contraceptives (LARCs), including hormonal implants and intrauterine devices are highly effective pregnancy prevention methods [1, 2]. LARCs are approximately 20 times as effective as other contraception methods, such as pills, patches, and rings [2]. Aside its advantages over other hormonal methods, LARCs are cost-effective, easy to maintain, and have a low risk of non-compliance-related method failure [1]. LARCs are also relatively safe for all sexually active women in the postpartum or postabortion period [2]. However, despite its effectiveness, most sexually active women use other short-term methods, such as condoms and contraceptive pills, which are associated with high discontinuation rates [2].

Although awareness and use of modern contraceptives have increased over the years [3], only about 15% of women globally use LARCs [4], with varying prevalence across regions, albeit, substantially lower in sub-Saharan Africa and Nigeria [5–7]. For example, evidence suggests that some 21.0% of sexually active women in Nigeria use traditional contraceptive methods , and only 14.8% use LARCs [7]. This indicates that a substantial percentage of sexually active women in Nigeria do not use LARCs, which may explain the very high total fertility rate (TFR) in the country as compared with other neighbouring African countries (5.2 TFR compared to 3.7 in Cameroon, 3.2 TFR in Ghana, and 2.2 TFR in Benin) [8, 9]. Importantly, high TFR is highly correlated to severe adverse consequences on maternal health outcomes, such as high maternal mortality ratios [10, 11]. It might be that complications increase as the number of children a woman has increases. Hence, the need to prioritise access to safe and effective family planning methods such as LARCs for sexually active women in Nigeria.

Studies have documented the importance of LARC over short-acting reversible contraceptive methods, including their efficiency and cost-effectiveness [12, 13]. However, there have been reports of discontinuation of LARCs due to several side effects, including irregular mensturation and delay in the return of fertility [14, 15]. Moreover, among sexually active women in sub-Saharan Africa, factors such as age, education, residence, knowledge of LARCs, being single, not having a child, partner’s education level, region, household wealth, employment status, religion, and community-level factors (e.g., literacy levels) influence LARC and contraception uptake generally [2, 16–18]. In addition to these factors, other health systems characteristics such as healthcare provider bias and cost [2, 19, 20] also act as barriers to LARCs use.

Considering the adverse effect of limited access to and underutilisation of LARCs on maternal health outcomes, it is important to understand factors predicting LARCs uptake in Nigeria using a robust spatial methodology. This is vital to improving the sexual and reproductive health outcomes of sexually active women in Nigeria. Moreover, there is a paucity of evidence on Nigeria’s spatial distribution and factors associated with LARC use. The importance of using spatial distribution tools is to identify the patterns of LARC use in Nigeria and its determinants. Therefore, this study examines the spatial distribution and multilevel factors associated with LARC use among sexually active women in Nigeria. Findings from this study have policy and public health implications to assist in prioritising efforts in locations and regions without or with suboptimal LARC uptake in Nigeria.

## Method and materials

### Data source

This is a cross-sectional analysis of a population-based study from the 2018 Nigeria Demographic Health Survey (NDHS). The NDHS is a nationally representative survey that collects datasets on socio-demographic characteristics, sexual and reproductive health-related indicators such as contraceptive use, and child & maternal health. The survey used a two-stage sampling procedure to collect data from 36 states and the Federal Capital Territory (FCT) in Nigeria. The samples were drawn randomly from clusters or enumeration areas (EAs) and served as the primary sampling unit for the survey. The 2018 survey included 41,821 women aged 15 to 49. However, 3978 sexually active women of reproductive age between 15 and 49 years who were either using an intrauterine device or implant in Nigeria met the inclusion criteria for the study. The NDHS sampling, pretesting, and general methodology have been published elsewhere [9, 21, 22]. We used a weighted sample size of reproductive-age women for the final analysis.

### Variables

#### Outcome variable

The study’s dependent variable was sexually active women currently using long-acting reversible contraceptives. Sexually active women of reproductive age in Nigeria who used one of the following long-acting contraceptive methods: intrauterine device or implant, are classified as LARC users [6, 7, 12, 18].

#### Explanatory variables

Individual and community-level variables were considered as independent variables in this study. Individual-level factors include respondent age, marital status, educational attainment, household wealth status, partner education, working status, terminated pregnancy, parity, fertility preference, religion, ethnicity, and access to mass media. At the same time, community literacy level, community socioeconomic status, residence, and region were the community-level factors [17, 23].

### Statistical analyses

#### Spatial analysis

To get the case to the total proportion, the weighted frequency of the outcome variable with cluster number was cross-tabulated in STATA software and exported to Excel. STATA 16 was used to combine geographic coordinate data. Observations within clusters with a longitude and latitude of zero were removed from the spatial analysis section. After that, the CSV file was imported into ArcGIS 10.7 for spatial analysis.

#### Spatial autocorrelation analysis

The spatial autocorrelation (Global Moran’s I) statistic determines whether LARC use patterns were dispersed, clustered, or randomly distributed in the study area. The output value of Moran’s I range from (-1) to + 1. Moran’s I value near 1 indicates disease dispersion, whereas Moran’s I value near + 1 indicates LARCs clustered and distributed randomly if Moran’s I is zero.

#### Hot spot analysis (Getis-Ord Gi* statistic)

Getis-OrdGi* statistical analysis was performed for each area to determine how spatial autocorrelation varies across the study area. The Z-score is computed to determine the statistical significance of clustering, and the p-value is computed. Statistical output with a high GI* indicates a “hotspot,“ whereas a low GI* implies a “cold spot.“ Hot spot areas indicated a high proportion of LARC use, while cold spot areas indicated low LARC use.

#### Spatial interpolation

Based on sampled EAs, the spatial interpolation technique is used to predict LARC use in un-sampled areas of the country. There are several methods for deterministic and geostatistical interpolation. The ordinary Kriging spatial interpolation method was used for this study to predict LARC use in unobserved areas of Nigeria, because it had a lower mean square error and residual than other interpolation techniques.

#### Spatial scan statistical analysis

Using Kuldorff SaTScanTM version 9.6 software, spatial scan statistical analysis was used to test for the presence of purely spatial low LARC use. Spatial scan statistics make use of a scanning window that moves across the study area. Women who used LARCs were treated as cases, while women who did not use LARCs were treated as controls. The dependent variable was assumed to be binary, and the Bernoulli assumption was used. As an upper limit, the default maximum spatial cluster size of 50 of the population was used, allowing both small and large clusters to be detected and clusters that contained more than the maximum limit to be ignored [24].

### Multilevel analysis

Binary logistic regression models with two levels of multilevel analysis were constructed to examine the individual and household/community level variables associated with LARC use among women of reproductive age in Nigeria. Women of reproductive age were nested within households and clusters to construct the models. Random effects were derived from the clusters in order to account for the unexplained variability at the community level. Four models were fixed in total. At first, an empty model representing “model 0” was fitted – this has no predictors (random intercept). After that, “model I” was constructed to contain only individual-level variables, while “model II” contained only household-level variables, and lastly, “model III” accounted for all the variables, which include both individual and household levels’ variables. We provided the odds ratio and corresponding 95% confidence intervals (CI) for models I to III. Stata command of “mlogit” was used to fit the models. The models were compared using the log-likelihood ratio and Akaike Information Criteria (AIC) measure [25]. The model with the highest log-likelihood and lowest AIC is the best fit [26]. The multicollinearity was also done using the variance inflation factor (VIF), and there was no evidence of collinearity among the independent variables. To account for the complex nature of the dataset, a population sample weight (v005/1000,000) was applied in all the analyses. We also use the “svy” command to account for the complex nature of the survey, which helps in the generalisation of the findings. The statistical analyses were done using STATA version 16.0 (Stata Corporation, College Station, TX, USA).

## Results

### Socio-demographic characteristics of sexually active women of reproductive age currently using long-acting reversible contraceptives in Nigeria

Out of 3978 respondents, about 2526 (63.52%) were living in urban areas, and 3,037 (76.36%) had secondary and higher education. The majority of respondents (85.22%) had media exposure, and 82.05% were employed. Almost all women (95.32%) were married, and 63.87% practised Christianity. The majority of the study participants were from the southwest, with 32.83% and 39.20% were from the richest household (Table 1).

**Table 1:** Sociodemographic characteristics of sexually active women of reproductive age currently using long-acting reversible contraceptives in Nigeria

Variables (n = 41,821)	Weighted frequency	Percent
Age		
15–24	424	10.66
25–34	1,757	44.17
35 and above	1,797	45.17
**Working status**		
No	714	17.95
Yes	3,264	82.05
**Marital status**		
Never married	187	4.68
Currently married	3,791	95.32
**Ever had terminated pregnancy**		
No	3,360	84.47
Yes	618	15.53
**Educational level**		
No Education	585	14.70
Primary	699	32.28
Secondary & above	2,694	67.72
**Husband education level**		
No Education	414	10.40
Primary	527	13.24
Secondary & above	3,037	76.36
**Total children ever born**		
No child	36	0.89
1–2 children	1,043	26.23
3–4 children	1,422	35.74
5 & above children	1,477	37.14
**Visited health facility last 12 months**		
No	1,982	49.83
Yes	1,996	50.17
**Fertility preference**		
Want more	2,087	52.46
Undecided	289	7.26
Want no more	1,602	40.28
**Media**		
No	588	14.78
Yes	3,390	85.22
**Religion**		
Christianity	2,540	63.87
Islam	1,431	35.99
Traditionalist & Others	6	0.15
**Ethnicity**		
Hausa	708	17.80
Yoruba	1,126	28.31
Igbo	864	21.71
Others	1,280	32.18
**Residence**		
Urban	2,526	63.52
Rural	1,452	36.48
**Wealth index**		
Poorest	218	5.48
Poorer	408	10.25
Middle	657	16.53
Richer	1,135	28.54
Richest	1,559	39.20
**Region**		
North central	557	14.01
North east	407	10.24
North west	620	15.59
South east	594	14.92
South-South	494	12.41
South west	1,306	32.83
**Sex of household head**		
Male	3,746	94.18
Female	232	5.82
**Community literacy level**		
Low	1,246	31.33
Medium	1,375	34.58
High	1,356	34.09
**Community socioeconomic status**		
Low	1,426	5.86
Medium	1,069	26.87
High	1,483	37.28

**NDHS, 2018**

### Spatial distribution of long-acting contraceptive use among sexually active women of reproductive age in Nigeria

**Fig. 1:**
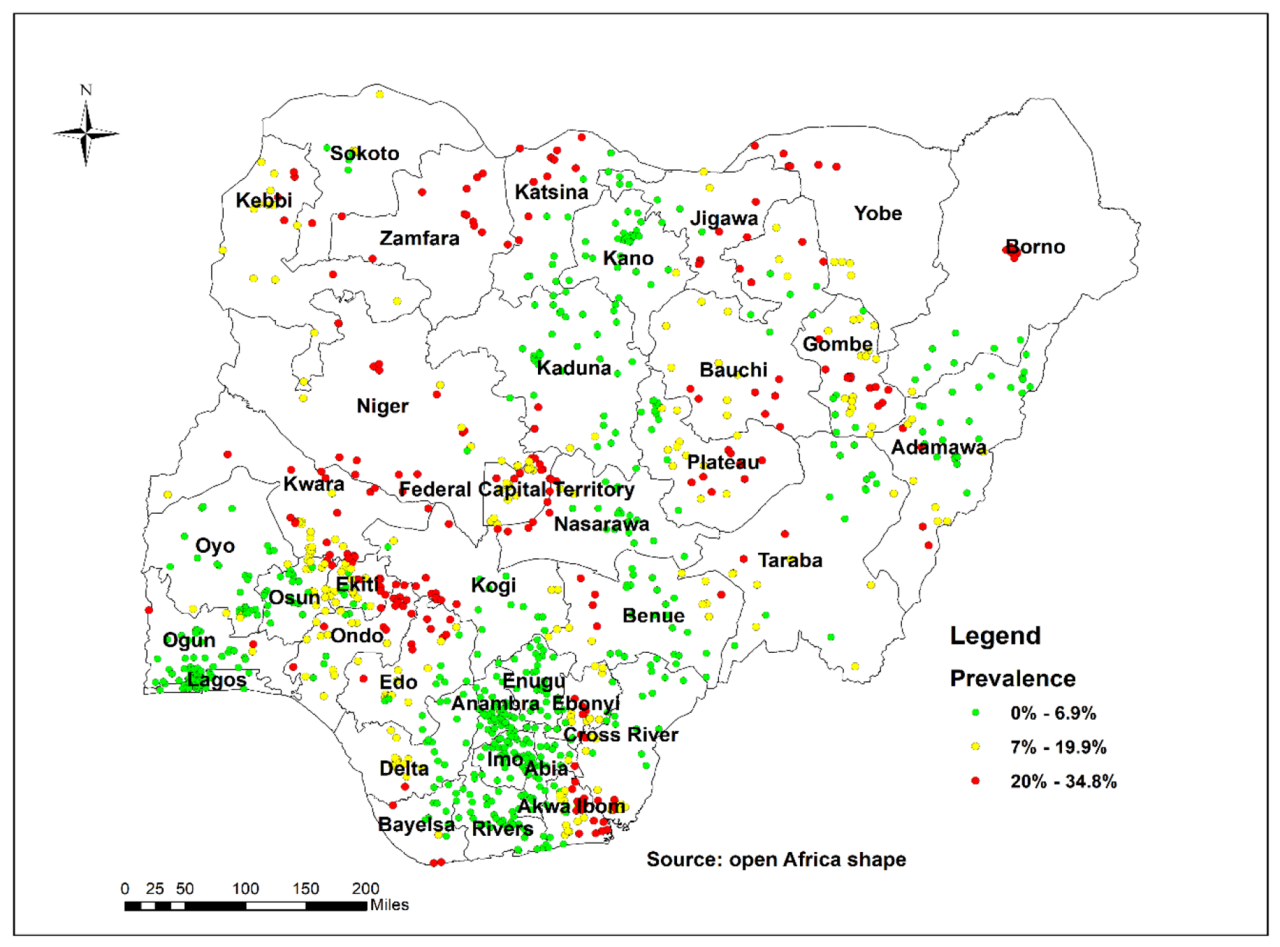
Spatial distribution of long-acting reversible contraceptive use among women of reproductive age in Nigeria (NDHS, 2018)

In Fig. 1, every point on the map represents an enumeration area, and each cluster’s proportion of LARC use cases is indicated. The red colour denotes areas with high proportions of LARC use ranging from 20 to 34.8%, while the green colour denotes areas with low proportions of LARC use ranging from 0 to 6.9%.

### Spatial autocorrelations of long-acting reversible contraceptive use among women of reproductive age in Nigeria

**Fig. 2:**
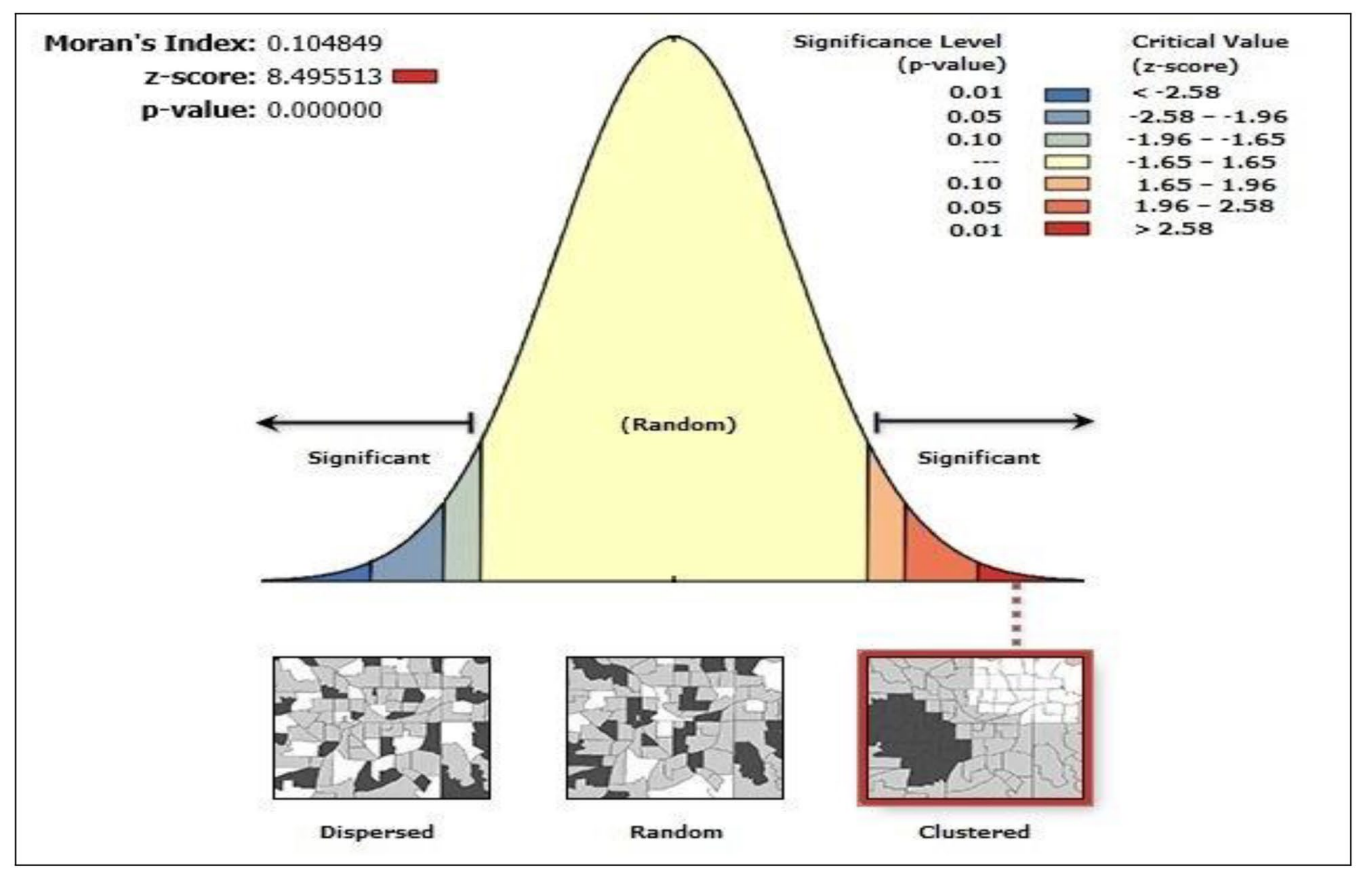
Spatial autocorrelation analysis of long-acting reversible contraceptive use among women of reproductive age in Nigeria (NDHS, 2018)

In the surveys, the spatial distribution of LARC varied by region in Nigeria. According to the spatial autocorrelation analysis results, LARC had spatial dependency in 2018 with (Moran’s I: 0.104, at P-value 0.001). This indicates that additional investigation is required to identify local-level clusters, as indicated in Fig. 2.

### Hotspot of long-acting reversible contraceptive use among women of reproductive age in Nigeria

**Fig. 3:**
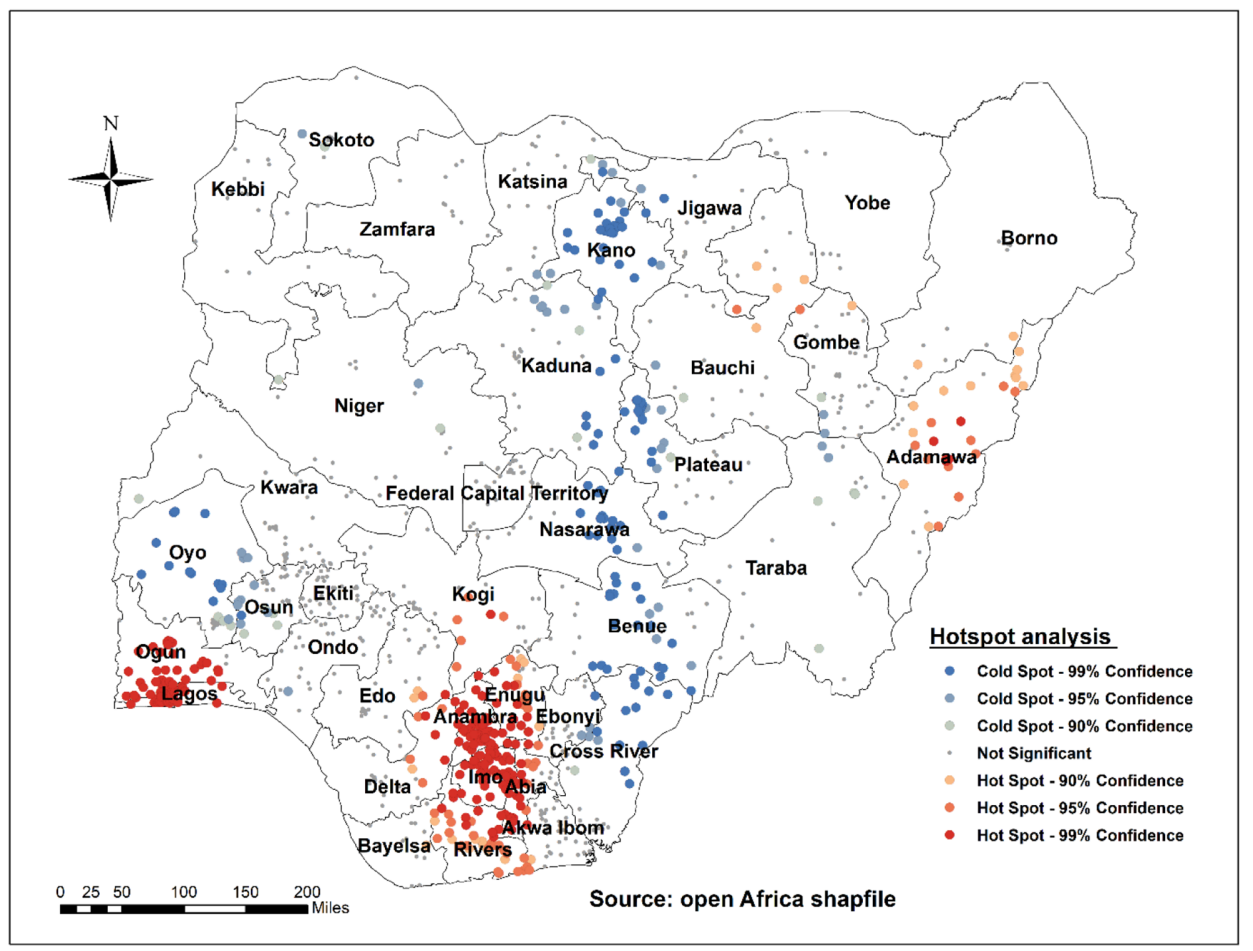
Hot Spot identification of long-acting contraceptive use among women of reproductive age in Nigeria (NDHS, 2018)

The hotspot analysis results show a low proportion (hotspot) and a high proportion (cold spot) of LARCs use in Nigeria. The red colours were found in the hotspot areas (low proportion of LARCs use) in Adamawa, Lagos, Ogun, Enugu, Anambra, Imo, Abia, and Rivers states. Cold spot (high proportion of LARCs use) areas were found in the states of Oyo, Osun, Kano, Kaduna, Plateau, Nasarawa, Benue, Cross River, and Jigawa (Fig. 3).

### Spatial interpolation of long-acting reversible contraceptive use among women of reproductive age in Nigeria

**Fig. 4:**
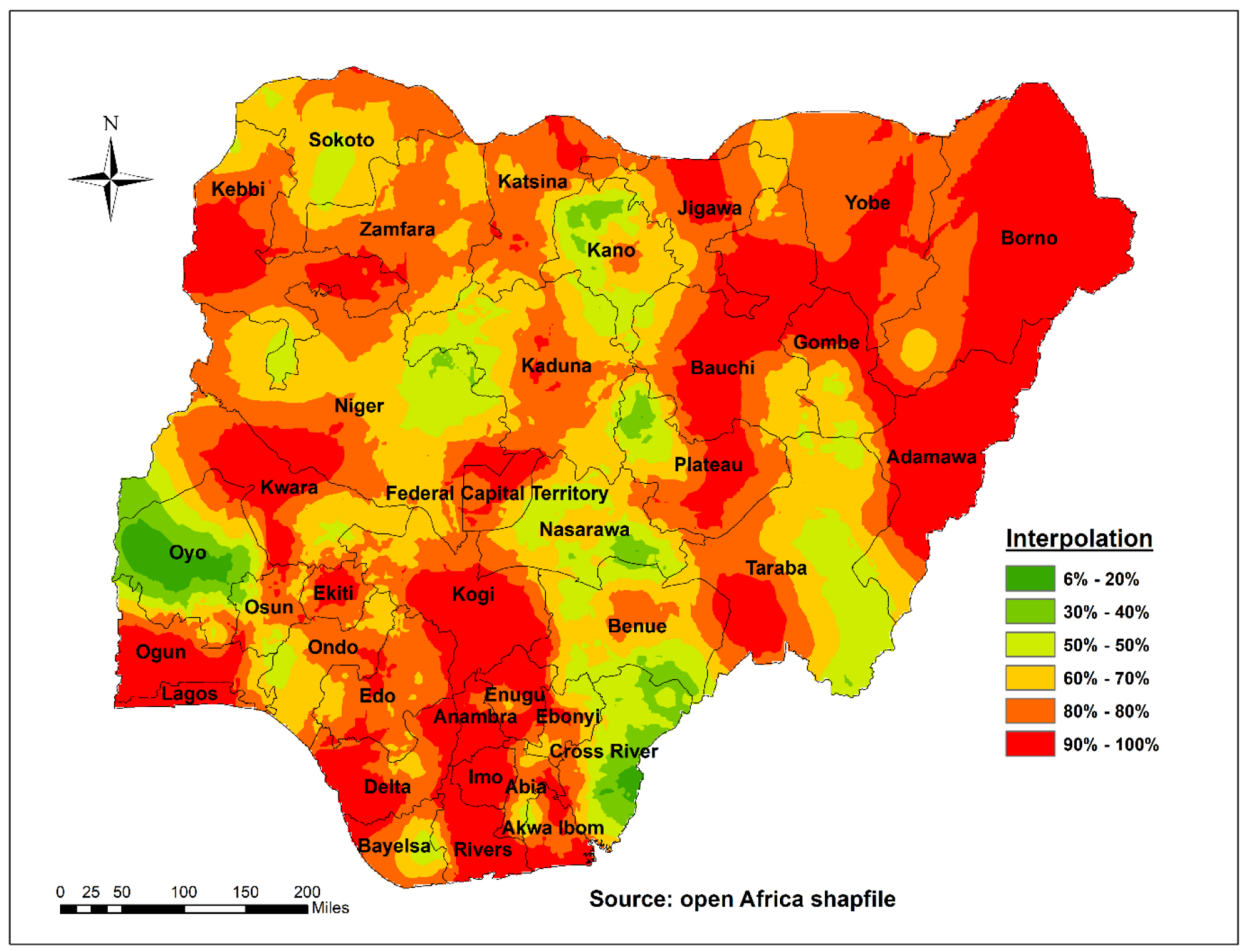
Spatial interpolation of long-acting reversible contraceptive use among women of reproductive age in Nigeria (NDHS, 2018)

The red colour in the spatial interpolation technique indicates the predicted low utilization of LARCs use in the country. As per the prediction results, Adamawa, Lagos, Ogun, Enugu, Anambra, Imo, Abia, Rivers, Kogi, Taraba, Yobe, Gombe, Jigawa, Borno, and Kebbi have a low proportion of LARC use. The Green colour prediction indicated that Oyo, Kano, Nasarawa, Cross Rivers, Benue, and Plateau had a high proportion of LARCs use in the country (Fig. 4).

### Multilevel analysis of factors associated with long-acting reversible contraceptive use among women of reproductive age in Nigeria

The current study employed a two-level mixed-effects logistic regression model to investigate the effects of individual and community-level factors on the use of long-acting contraceptives. Abortion, educational level, fertility preference, region, and community socioeconomic status were factors associated with the use of long-acting contraceptives in Nigeria.

In this study, women with a prior history of abortion had a 38% (aOR = 0.62, 95% CI: 0.48–0.80) lower likelihood of using LARCs than their counterparts. The odds of using long-acting contraceptive methods among women with secondary education & above were 1.63 times higher (aOR = 1.63, % CI: 1.17–2.26) compared with women with no formal education. In terms of fertility preference, women who did not want more children were 1.65 times (aOR = 1.65, 95% CI: 1.30–2.08) more likely to use long-acting contraception than women who wanted more children. At community socioeconomic status, women from higher socioeconomic status communities had 34% (aOR = 0.66; % CI: 0.45–0.97) lower odds of using LARCS when compared to their counterparts, as shown in Table 2.

**Table 2:** Multilevel logistics regression model for individual and household/community-level factors associated with long-acting reversible contraceptive use among sexcually active women of reproductive age in Nigeria

Variables	Model 0	Model I AOR [95%CL]	Model II AOR [95%CL]	Model III AOR [95%CL]
Age				
15–24		1		1
25–34		0.85(0.61–1.18)		0.84(0.61–1.18)
35 and above		0.87(0.59–1.27)		0.86(0.59–1.26)
Working status				
Yes		1.28(1.00-1.64)		1.28(1.00-1.63)
No		1		1
**Marital status**				
Never married		1		1
Currently married		1.08(0.69–1.68)		0.98(0.63–1.52)
**Pregnancy termination**				
Yes		0.63(0.49–0.81) **		0.62(0.48–0.80) **
No		1		1
**Educational level**				
No formal education		1		1
Primary education		1.37(0.99–1.90)		1.37(0.99–1.90)
Secondary education & above		1.59(1.16–2.18) **		1.63(1.17–2.26) **
**Husband education level**				
No Education		1		1
Primary		0.94(0.64–1.38)		0.95(0.65–1.39)
Secondary education & above		1.03(0.73–1.46)		1.00(0.71–1.41)
**Total children ever born**				
No child		1		1
1–2 children		9.66(1.18–78.7)		9.47(1.17–76.5)
3–4 children		13.2(1.61–108.6)		12.7(1.56–103.8)
5 & above children		15.4(1.86–127.5)		14.8(1.80-121.8)
**visited health facility last 12 months**				
no		1		1
yes		1.12(0.93 1.33)		1.08(0.90–1.29)
**fertility preference**				
want more		1		1
Undecided		1.46(1.01–2.09)		1.48(1.04–2.12)
want no more		1.62(1.29–2.05) **		1.65(1.30–2.08) **
**Media**				
No		1		1
Yes		1.07(0.83–1.38)		1.11(0.85–1.44)
**Religion**				
Christianity		1		1
Islam		0.84(0.65–1.09)		0.74(0.57–0.97)
Traditionalist & Others		1.65(0.36–7.57)		2.50(0.56–10.9)
**Ethnicity**				
Hausa		1		1
Yoruba		0.67(0.46–0.97)		1.14(0.70–1.87)
Igbo		0.34(0.22–0.52) **		0.97(0.56–1.69)
Others		0.79(0.56–1.11)		1.27(0.86–1.89)
**Household/Community level**				
**Residence**				
Urban			1	1
Rural			0.82(0.64–1.04)	0.77(0.60–0.98)
**Wealth index**				
Poorest			1	1
Poorer			0.97(0.65–1.45)	0.89(0.59–1.34)
Average			1.05(0.71–1.57)	0.97(0.64–1.47)
Richer			1.05(0.69–1.60)	0.99(0.63–1.55)
Richest			1.08(0.67–1.74)	1.00(0.60–1.67)
**Geopolitical Zone**				
North-Central			1	1
North-East			0.52(0.37–0.75) **	0.71(0.49–1.03)
North-West			1.21(0.86–1.69)	2.07(1.36–3.16) **
South-East			0.33(0.22–0.47) **	0.32(0.19–0.53) **
South-South			0.63(0.44–0.90)	0.56(0.38–0.81) **
South-West			0.66(0.49–0.90) **	0.68(0.46-1.00)
**Sex of household head**				
Male			1	1
Female			0.92(0.65–1.30)	0.97(0.68–1.37)
**Community literacy level**				
Low			1	1
Medium			1.12(0.86–1.47)	0.99(0.75–1.31)
High			1.21(0.90–1.64)	1.05(0.76–1.44)
**Community socioeconomic status**				
Low			1	1
Medium			0.86(0.63–1.16)	0.84(0.62–1.14)
High			0.64(0.44–0.94)	0.66(0.45–0.97) *
PSU Variance (95% CI)	0.96[0.70–1.32]	0.84[0.59–1.19]	0.71[0.49–1.04]	0.67[0.45–0.99]
ICC	0.23	0.20	0.18	0.17
LR Test	χ2 = 108.17 p < 0.001	χ2 = 80.24, p < 0.001	χ2 = 62.96, p < 0.001	χ2 = 53.19, p < 0.001
Wald χ2	Reference	114.52***	62.79***	165.05***
**Model fitness**				
Log-likelihood	-2075.31	-2010.97	-2044.33	-1982.74
AIC	4154.62	4067.93	4122.66	4041.48
Number of clusters	1042	1042	1042	1042

*Weighted NDHS, 2018*

*Exponentiated coefficients; 95% confidence intervals in brackets; AOR = adjusted Odds Ratios; CI = Confidence Interval; RC = Reference Category*

*p < 0.05; **p < 0.01; ***p < 0.001

*PSU = Primary Sampling Unit; ICC = Intra-Class Correlation; LR Test = Likelihood ratio Test; AIC = Akaike’s Information Criterion*

*Model 0 is the null model, a baseline model without any determinant variable*

*Model I include individual-level variables (Age of respondent, working status, marital status, educational level, husband educational level, total children ever born, visited health facility last 12 months, fertility preference, media, religious, ethnicity)*

*Model II include household/community level variables (Place of residence, wealth index, region, sex of household head, community literacy level, and community socioeconomic status)*

*Model III the final model included both individual and household/community level variables*

### Random effects (measures of variations) of factors associated with long-acting reversible contraceptive use among sexually active women of reproductive age in Nigeria

The empty model (Model 0), as shown below in Table 2, depicted a variation in the likelihood of LARCs use among sexually active women of reproductive age in Nigeria across the Primary Sampling Units (PSUs) clustering [σ2 = 0.96; 95%(CI = 0.70–1.32)]. The Model 0 indicated that 23% of the variation in LARCs use among sexually active women of reproductive age in Nigeria was attributed to the variation between Intra-Class Correlation, i.e., (ICC = 0.23). The variation between-cluster decreased to 17% (0.17) in Model III (individual and household/community factors). This reiterates that the variations in the likelihood of LARCs use among sexually active women of reproductive age in Nigeria are attributed to the clustering variation in PSUs. Therefore, Model III, the complete model with both the selected individual and household/community factors, was selected to predict the likelihood of LARCs use among sexually active women of reproductive age in Nigeria.

## Discussion

This study was motivated by a need to better understand national patterns in LARCs use to further improve and promote reproductive and overall health and well-being of sexually active women in Nigeria. Within the larger sub-Saharan African setting, evidence has shown that short-term contraceptives seem to be the most commonly used type of contraceptives [27, 28], and these methods may have a high risk of non-compliance related failures [2]. This thus necessitates understanding how LARCs––with a lower risk of consistency-related failure––﻿can be improved among sexually active women of reproductive age in a country such as Nigeria, which has a high fertility rate.

Findings from our spatial interpolation technique suggest low utilisation of LARCs in Nigeria as most parts of the country were found to be hotspots (i.e., areas with low-level of LARCs utilisation). This low level of LARCs utilisation is in line with the findings of previous studies that have reported low utilisation of LARCs in Nigeria [7] and other sub-Saharan African countries [6, 16, 29, 30] partly because of preferences for short-acting contraceptives [31] or even the traditional methods [7]. Notably, parts of Nigeria, which may be considered more cosmopolitan, such as Lagos, Enugu, Ogun, and Rivers states, were also found to be hotspot areas with low utilisation of LARCs. Considering that most participants were selected from urban areas and a significant proportion from settings where cost may not be an issue with accessing LARCs, there may be a need to look closely into setting-specific factors that may influence LARCs acceptability utilisation in Nigeria.

In the present study, we found that women with a history of abortion were less likely to use LARCs. This could be related to the likelihood of choosing to resort to abortion, should an unplanned pregnancy occur. Considering that abortion is largely illegal in Nigeria there may be high chances of engaging in crude and unsafe abortion methods and/or engaging the services of quacks, a practice attributed to adverse health outcomes for reproductive-age women in Nigeria [32]. Therefore, women with a history of abortion may constitute a sub-population of reproductive age women to be targeted with interventions to increase LARCs use.

Furthermore, in support of previous findings [6, 16, 33], we found that women with secondary education or above are more likely to use LARCs in comparison with those with lower or no formal education. Women who are educated are more likely to possess health literacy skills, and therefore, they are more able to seek contraceptive information and make better-informed decisions, including the use of LARCs. In the same vein, they are also more likely to be engaged in the formal work sector, which could motivate them to use reliable and convenient contraceptive methods such as LARCs to avoid frequent pregnancy-related disruptions to their careers. These circumstances may not apply to women without formal education, who may also be more likely to work in non-formal sectors and may therefore be less motivated to seek out long-lasting contraceptive options. With appropriate interventions, the utilisation of LARCs can be increased among women with or without formal education. This is especially the case for the latter, who have shown high unmet contraceptive needs [33].

Another reason for low utilisation of LARCs could be due to provider dependence, which requires that users are obligated to visit healthcare services for insertion and remover of these devices [16]. Other reasons include perceived delay in the return of fertility, provider bias and menorrhagia [15]. Women may also consider using the newly introduced depot medroxyprogesterone acetate (DMPA-SC) over IUD & implant methods because of the advantage of self-administration as it doesn’t involve provider dependence [34].

Additionally, as expected, we found, and in line with previous studies [12, 33], that LARC use is higher among women who do not intend for more children than their counterparts. Reaching women with future pregnancy intentions with accurate information and message about the reversibility of LARCs could be an important strategy for increasing LARCs use among this sub-population. LARCs are relevant and appropriate for women with future pregnancy intention for appropriate child spacing, which is vital for improving and promoting maternal and child health care [35, 36].

### Strengths and Limitations

Data used in this study are from highly reliable and valid sources and are nationally representative of the geo-political zones in Nigeria. Consequently, conclusions drawn can be generalised with a higher sense of confidence. Moreover, by deploying geographical information systems in analysing the spatial distribution of LARCs use, this study contributes to the literature and provides insights into geo-political zones and states in Nigeria that are hotspots with low LARCs use and where interventions to increase LARCs can be focused. However, the study’s findings need to be understood within some caveats. Notably, data for the study were based on self-reports and are, therefore, subject to social desirability responses and recall bias. Another limitation is that the NDHS 2018 did not capture the location of LARC services; hence, the spatial distribution may reflect available LARC services. Also, due to the nature of the data, aside from association, causality cannot be established. Again, some context-specific factors that would have enhanced understanding of LARC use cannot be investigated as such measures are not present in the dataset. The present study thus makes a unique contribution to identifying these critical blind spots for future studies to explore.

### Policy and practical implications

The results of this study have important implications for both policy and practice. There is a need for an educational policy review to achieve long-term maternal and child health goals, using education as a tool. The need for educating the girl child cannot be overemphasised. Beyond enrolment, policies to retain enrolled women of reproductive age, provide them with functional education, and assimilate them into the formal sector of the economy should be pursued. This is necessary as educated and employed women are more empowered and motivated to make informed decisions about their reproductive health. In the same vein, it is important to revisit policies around maternal and child health services across all levels of the Nigerian healthcare system to mandate educating and counseling women of reproductive age on family planning choices. These policies should encourage further training of health workers on providing LARC services to women of reproductive age in Nigeria, and adequate and proper funding should be allocated for these projects by the government and responsible non-governmental organisations. These policies, when translated into practice, would ensure that every woman of reproductive age that accesses any healthcare system, for whatever reasons, is provided with information about her reproductive health and rights and how to make informed decisions about safe motherhood.

## Conclusion

This study showed low utilisation of LARCs among sexually active reproductive-age women in Nigeria. This low utilisation is also common in states that could be described as cosmopolitan, indicating a need for closer investigation to understand context-specific factors associated with LARC use. Women without formal education and/or primary education constitute an important sub-population-group that needs greater attention in designing tailored interventions to improve LARC use. Population-specific family planning education and counseling for this group are important to address misconceptions that may be common about LARCs in particular and modern contraceptive use in general.

## Data Availability

The datasets utilized in this study can be accessed at https://dhsprogram.com/data/available-datasets.cfm.

## Abbreviations

LARCs: Long-acting Reversible Contraceptives
NDHS: Nigeria Demographic Health Survey
WHO: World Health Organisation
TFR: Total Fertility Rate
FCT: Federal Capital Territory
EAs: Enumeration Areas
CI: Confidence Intervals
AIC: Akaike Information Criteria
VIF: Variance Inflation Factor.
